# Molecular characterization of livestock-associated ticks and tick-borne bacteria in Xinjiang, northwestern China

**DOI:** 10.1186/s13071-025-07178-z

**Published:** 2025-12-02

**Authors:** Haipeng Tan, Xiaonan Dong, Jiamei Kang, Nan Bu, Yishuai Zhang, Zehao Qi, Zixuan Li, Zilong Zhang, Xuyang Zhang, Huidong Wang, Yulin Ding, Yonghong Liu, Li Zhao

**Affiliations:** 1 College of Veterinary Medicine, Inner Mongolia Agricultural University, Hohhot, China; 2 Key Laboratory of Clinical Diagnosis and Treatment Technology in Animal Disease, Ministry of Agriculture and Rural Affairs, Hohhot, China

**Keywords:** Tick, *Rickettsia*, *Anaplasma*, *Borrelia*, Livestock, Xinjiang, China

## Abstract

**Background:**

Xinjiang Uygur Autonomous Region represents a critical pastoral zone at the livestock–tick–human interface in northwestern China, yet molecular data on tick-borne pathogens in this region remain scarce.

**Methods:**

Between 2017 and 2018, 6172 ticks were collected from cattle, sheep, goats, horses, and dogs across 18 counties in Xinjiang. Tick species identification was performed through morphological examination and cytochrome oxidase I (*COI*) gene barcoding. Pooled samples (*n* = 55) were screened using polymerase chain reaction (PCR) and sequencing targeting *Rickettsia* (glutamate transporter A [*gltA*], outer membrane protein A [*ompA*] genes), *Anaplasma* (16S ribosomal RNA [*16S rRNA*]), *Borrelia* (heat shock protein GroEL [*groEL*]), and broad-range bacterial diversity (*16S rRNA*).

**Results:**

Seven tick species were identified: *Alveonasus lahorensis* (33.7%), *Dermacentor marginatus* (32.3%), *Rhipicephalus turanicus* (21.9%), *Dermacentor silvarum* (5.7%), *Hyalomma asiaticum* (4.0%), and *Haemaphysalis sulcata* (2.5%). *Rickettsia* DNA was detected in 28 of 55 pools (50.9%), with sequences showing relatedness to *Rickettsia raoultii*, *Rickettsia massiliae*, and *Rickettsia barbariae*. *Anaplasma capra* was identified in *D. marginatus* collected from goats (1.8% of pools), while *Borrelia miyamotoi* was detected in *R. turanicus* from sheep (1.8% of pools). Additional bacterial genera detected included *Arsenophonus* in *D. marginatus*, *Coxiella* in *R. turanicus*, and *Francisella* in *H. asiaticum*. Notably, *R. massiliae* was detected in both eggs and unfed larvae of *R. turanicus*, consistent with transovarial transmission.

**Conclusions:**

This study represents the first comprehensive molecular survey of livestock-associated ticks in Xinjiang, revealing high prevalence of spotted fever group rickettsiae and the presence of emerging tick-borne pathogens. Our findings underscore potential zoonotic risks within pastoral systems and emphasize the critical need for enhanced One Health surveillance strategies at the livestock–human interface in this region.

**Graphical Abstract:**

**Figure:**
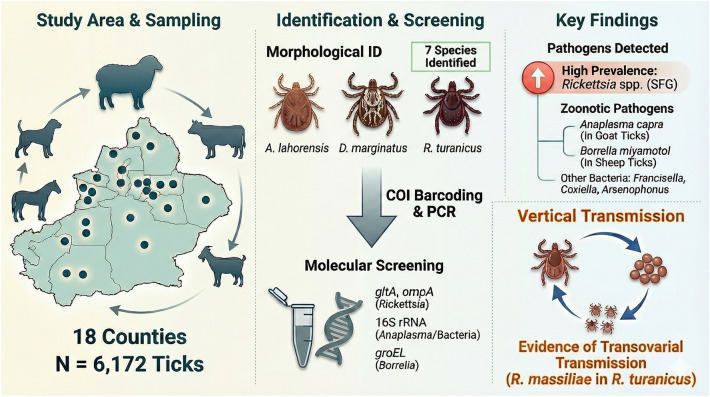

## Background

Ticks are obligate blood-feeding ectoparasites of significant medical and veterinary importance, serving as vectors for a diverse array of bacterial, viral, and protozoan pathogens that cause serious diseases in humans and animals [1]. In recent decades, global warming, ecological changes, and intensified interactions among wildlife, livestock, and humans have accelerated the emergence and spread of tick-borne diseases worldwide [2]. China harbors more than 120 tick species, yet molecular surveillance of tick fauna and associated pathogens remains geographically uneven [3]. While tick species and their associated pathogens have been relatively well studied in northeastern provinces such as Heilongjiang and Inner Mongolia, research in northwestern pastoral regions, including the Xinjiang Uygur Autonomous Region, remains relatively scarce. This gap in knowledge may hinder effective regional public health preparedness and response [4].

The Xinjiang Uygur Autonomous Region represents a particularly critical ecological and epidemiological setting for tick-borne disease research. As China’s largest pastoral region, Xinjiang borders eight countries and sustains extensive populations of cattle, sheep, goats, horses, and dogs that experience heavy tick exposure [5]. The region’s strategic geographical position at the crossroads of Central Asia suggests that local tick populations and associated pathogens may be influenced by transboundary ecological and epidemiological processes [6]. While infections with spotted fever group *Rickettsia* and novel *Anaplasma* species have been documented in Xinjiang, comprehensive molecular surveys remain scarce. Evidence from neighboring Central Asian (including countries such as Kazakhstan, Kyrgyzstan, Uzbekistan, and Tajikistan) countries indicates substantial pathogen diversity, including *Borrelia* and emerging *Anaplasma* species, underscoring the urgent need for systematic assessments within Xinjiang [7, 8].

Several important knowledge gaps persist regarding ticks and their associated pathogens in this region. Sequence-based confirmation of tick species across multiple counties remains limited, simultaneous screening for multiple bacterial genera has rarely been undertaken, and the potential for vertical transmission of rickettsiae in local tick populations has not been systematically investigated. To address these research needs within a One Health framework, we conducted a comprehensive molecular survey of livestock-associated ticks across 18 counties in Xinjiang. Our primary objectives were to characterize tick species composition using morphological identification and cytochrome oxidase I*COI* barcoding, to detect major bacterial pathogens (*Rickettsia*, *Anaplasma*, and *Borrelia*) through polymerase chain reaction (PCR) amplification and phylogenetic validation, to assess broader bacterial diversity using 16S ribosomal RNA (rRNA) gene sequencing, and to investigate potential vertical transmission patterns of *Rickettsia* species, as done for other objectives using PCR screening of egg masses and unfed larvae from *Rhipicephalus turanicus* females.

## Methods

### Study area and sample collection

**Fig. 1:**
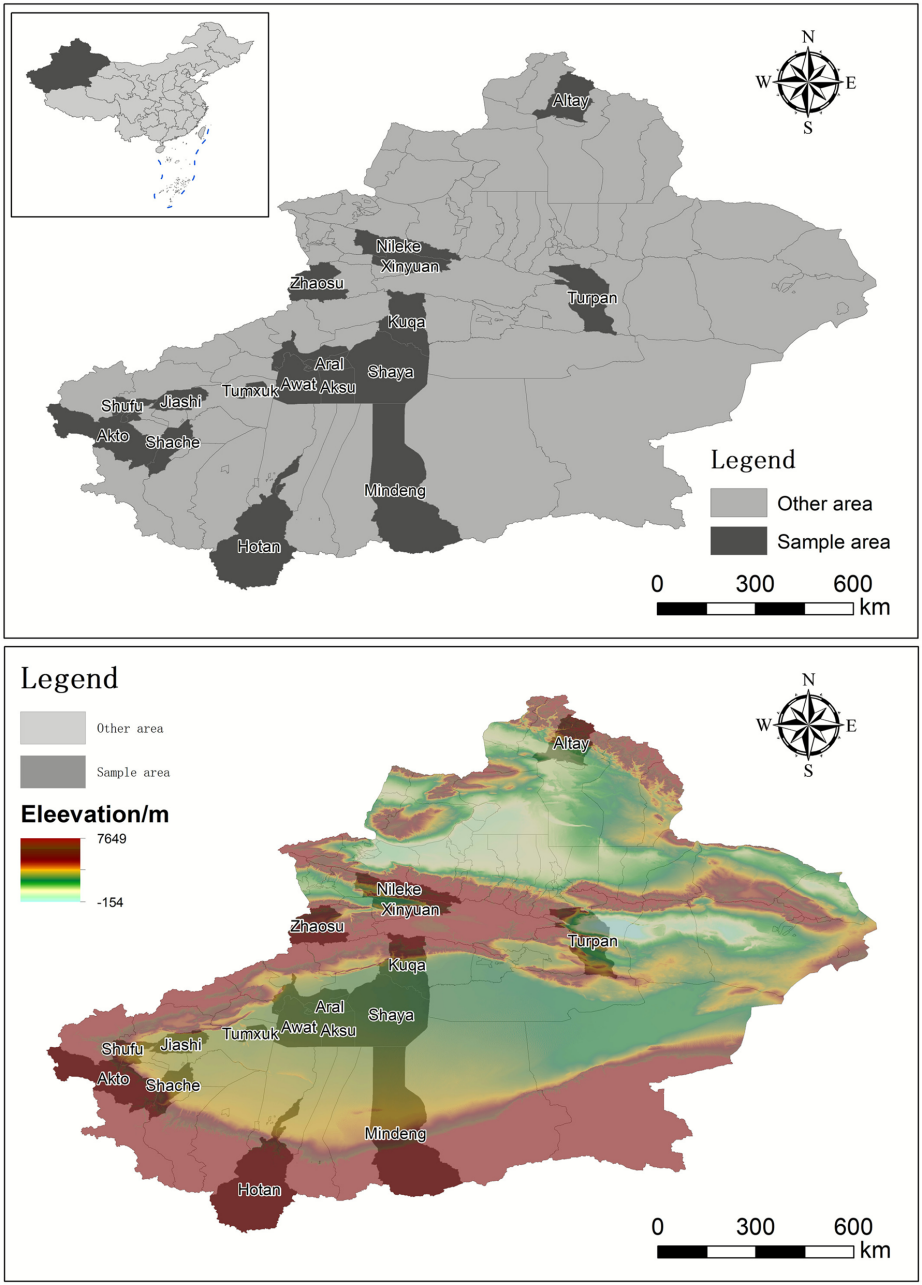
Sampling sites across the Xinjiang Uygur Autonomous Region. The base map was created using ArcGIS 10.8 software with administrative boundary data obtained from the National Geomatics Center of China (http://www.ngcc.cn/)

An ecological survey was conducted from 2017 to 2018 across the Xinjiang Uygur Autonomous Region (40° 25′–49° 10′ N, 73° 25′–96° 23′ E), employing a multistage stratified sampling design. A total of 18 ecologically representative counties were selected to capture the region’s diverse pastoral environments, including Aral, Hotan, Altay, Akto, Kuqa, Minfeng, Awat, Aksu, Shaya, Tumxuk, Turpan, Shufu, Jiashi, Kashgar, Shache, Xinyuan, Zhaosu, and Nileke (Fig. 1). The map was generated using ArcGIS 10.8 on the basis of the administrative boundary data obtained from the National Geomatics Center of China (http://www.ngcc.cn). Sampling targeted livestock species (cattle, sheep, goats, and horses) and companion animals (dogs) on the basis of their epidemiological relevance for tick-borne pathogen transmission.

To ensure adequate statistical power, sample size requirements were estimated on the basis of an expected pathogen prevalence of 10–30% reported in Central Asian tick surveillance studies [9]. Using 80% power and 95% confidence, the minimum sample size was calculated as 384 ticks per major species group. In practice, however, we aimed to maximize coverage across hosts and sites, and ultimately obtained 6172 adult ticks by whole-body examination of livestock during peak activity (April to September). Detailed information on sampling sites, tick species collected, and pooling strategy is presented in Table 1.

**Table 1:** Counts of tick species collected from the sampling sites around XinJiang during 2017 and 2018

No. of sampling sites	County	Host	Tick species	Tick no.	Pooled tick group
1	Aral	Sheep	*R. turanicus*	323	1
		Dogs	*R. turanicus*	3	2
2	Hotan	Sheep	*A. lahorensis*	54	3
		Cattle	*H. sulcata*	153	4
3	Altay	Cattle	*D. marginatus*	58	5
4	Akto	Sheep	*R. turanicus*	57	6
		Sheep	*A. lahorensis*	39	7
5	Kuqa	Sheep	*A. lahorensis*	86	8
		Sheep	*A. lahorensis*	55	9
		Sheep	*A. lahorensis*	39	10
		Sheep	*A. lahorensis*	116	11
		Sheep	*A. lahorensis*	148	12
		Sheep	*A. lahorensis*	96	13
		Sheep	*R. turanicus*	63	14
		Cattle	*D. marginatus*	109	15
6	Minfeng	Sheep	*A. lahorensis*	57	16
7	Awat	Dogs	*R. turanicus*	54	17
		Dogs	*R. turanicus*	39	18
		Sheep	*R. turanicus*	125	19
		Dogs	*R. turanicus*	7	20
		Goat	*R. turanicus*	131	21
		Sheep	*R. turanicus*	71	22
8	Aksu	Dogs	*R. turanicus*	14	23
		Dogs	*R. turanicus*	9	24
9	Shaya	Goat	*D. marginatus*	283	25
		Goat	*H. asiaticum*	247	26
		Goat	*D. marginatus*	79	27
10	Tumxuk	Cattle	*R. turanicus*	37	28
11	Turpan	Sheep	*A. lahorensis*	99	29
12	Shufu	Sheep	*A. lahorensis*	82	30
		Sheep	*A. lahorensis*	47	31
13	Jiashi	Sheep	*A. lahorensis*	150	32
		Sheep	*A. lahorensis*	60	33
		Sheep	*A. lahorensis*	110	34
		Sheep	*R. turanicus*	245	35
14	Kashgar	Sheep	*R. turanicus*	35	36
		Sheep	*A. lahorensis*	79	37
		Sheep	*R. turanicus*	75	38
		Sheep	*A. lahorensis*	46	39
		Sheep	*A. lahorensis*	54	40
		Sheep	*A. lahorensis*	80	41
		Sheep	*A. lahorensis*	80	42
		Sheep	*A. lahorensis*	60	43
15	Shache	Cattle	*R. turanicus*	63	44
16	Xinyuan	Sheep	*D. marginatus*	30	45
		Horse	*D. marginatus*	300	46
		Sheep	*D. marginatus*	280	47
		Sheep	*A. lahorensis*	100	48
		Sheep	*D. silvarum*	72	49
		Sheep	*D. marginatus*	137	50
		Sheep	*D. silvarum*	280	51
		Sheep	*D. marginatus*	160	52
17	Zhaosu	Sheep	*D. marginatus*	180	53
18	Nileke	Sheep	*D. marginatus*	376	54
		Sheep	*A. lahorensis*	340	55

^a^ Each sample group contributed one pool of six adult ticks for pathogen screening

### Morphological and molecular species identification

Morphological identification was performed using an Olympus BX53 optical microscope at 400× magnification following standard taxonomic keys [10, 11]. For molecular validation, 10% of specimens per morphologically identified species (*n* = 617) underwent COI barcoding. Genomic DNA was extracted with the AllPrep DNA/RNA Mini Kit (QIAGEN, Shenzhen, China), and COI fragments (658 base pairs [bp]) were amplified (primers presented in Table 2). Amplicons were sequenced bidirectionally and analyzed for phylogenetic confirmation.

**Table 2:** Primers for ticks and tick-borne pathogen detection

Organism	Gene name	Primer name	Primer sequence 5′ to 3′	Approximate amplicon/bp
Ticks	*COI*	Tick *COI*-F	GTTCAACAAATCATAAAGATATTGG	658 bp
		Tick *COI*-R	TAAACTTCAGGGTGACCAAAAAATCA	
*Rickettsia* spp.	*gltA*	R-*gltA*-F	ATGACCAATGAAAATAATAAT	1060 bp
		R-*gltA*-R	ATTGCAAAAAGTACAGTGAACA	
	*ompA*	R-*ompA*-F	ATGGCGAATATTTCTCCAAAA	491 bp
		R-*ompA*-R	AGTGCAGCATTCGCTCCCCCT	
*Anaplasma* spp.	*16S rRNA*	AC-F	GGTACCYACAGAAGAAGTCC	344 bp
		AC-R	TAGCACTCATCGTTTACAGC	
*Borrelia* spp.	*groEL*	Bo-F	TACGATTTCTTATGTTGAGGG	310 bp
		Bo-R	CATTGCTTTTCGTCTATCACC	
Multiple bacterial	*16S rRNA*	Ba-F	CTAHAGGGTATCTAATCCT	789 bp
		Ba-R	GAGTTTGATCMTGGCTCAG	

### Pooling strategy and nucleic acid extraction

For pathogen detection, ticks were pooled by species, host, site, stage, and date. Each pool contained six individuals, consistent with pooling strategies employed in similar tick-borne pathogen surveys [12]. A total of 55 pools (330 ticks) were prepared.

Ticks were surface-sterilized in 5% bromogeramine, 75% ethanol, and phosphate-buffered saline (PBS) (15 min each) and then air-dried. Specimens were homogenized under sterile conditions. DNA was extracted using the AllPrep DNA/RNA Mini Kit and eluted in 100 μL. Extracts were stored at −20 °C.

### PCR amplification and quality control

Target genes included *Rickettsia* (*gltA*, *ompA*), *Anaplasma* (*16S rRNA*), *Borrelia* (*groEL*), and broad-range bacterial *16S rRNA* (Table 2). Each PCR batch contained positive controls (reference DNA), negative controls (PCR-grade water), and extraction blanks. Laboratory workflow strictly separated pre- and post-PCR areas to avoid contamination.

PCR reactions were 25 μL with 2 × Master Mix, 10 μM primers, and 2 μL template DNA. Thermal cycling conditions were optimized for each target gene through preliminary experiments. Optimization included testing annealing temperatures (50–60 °C), extension times (30–90 s), and cycle numbers (30–40 cycles) to ensure specific amplification efficiency. Products were visualized on 1.5% agarose gels and positive amplicons sequenced on an ABI 3730xl platform.

### Sequence analysis and phylogenetic reconstruction

Sequences were trimmed, assembled in SeqMan Pro (DNASTAR), and compared with GenBank references using Basic Local Alignment Search Tool (BLAST). Multiple sequence alignments were generated in MEGA X using ClustalW algorithm [13]. Phylogenetic relationships were inferred using the neighbor-joining method with Kimura 2-parameter model. Nodal support was assessed with 1000 bootstrap replicates, and trees were rooted using appropriate outgroup sequences [14].

### Statistical analysis

Pool positivity (proportion of positive pools) was calculated with 95% Wilson confidence intervals. Minimal infection rates (MIRs) were estimated where appropriate using the formula: MIR = (positive pools × 100)/total ticks tested [15]. Associations between pathogen detection and tick species, host animals, or geographic locations were evaluated using *χ*^2^ tests or Fisher’s exact tests where cell counts were < 5. Statistical significance was set at *α* = 0.05. All analyses were performed using R software, version 4.2.0.

### Vertical transmission investigation

To investigate potential vertical transmission, a subset of *R. turanicus* females collected from cattle in Kashgar were maintained in laboratory conditions. Eggs were collected aseptically and allowed to develop into unfed larvae. Both egg masses and newly hatched larvae were processed for DNA extraction and PCR screening using the same protocols described above.

## Results

### Tick species composition and host associations

**Fig. 2:**
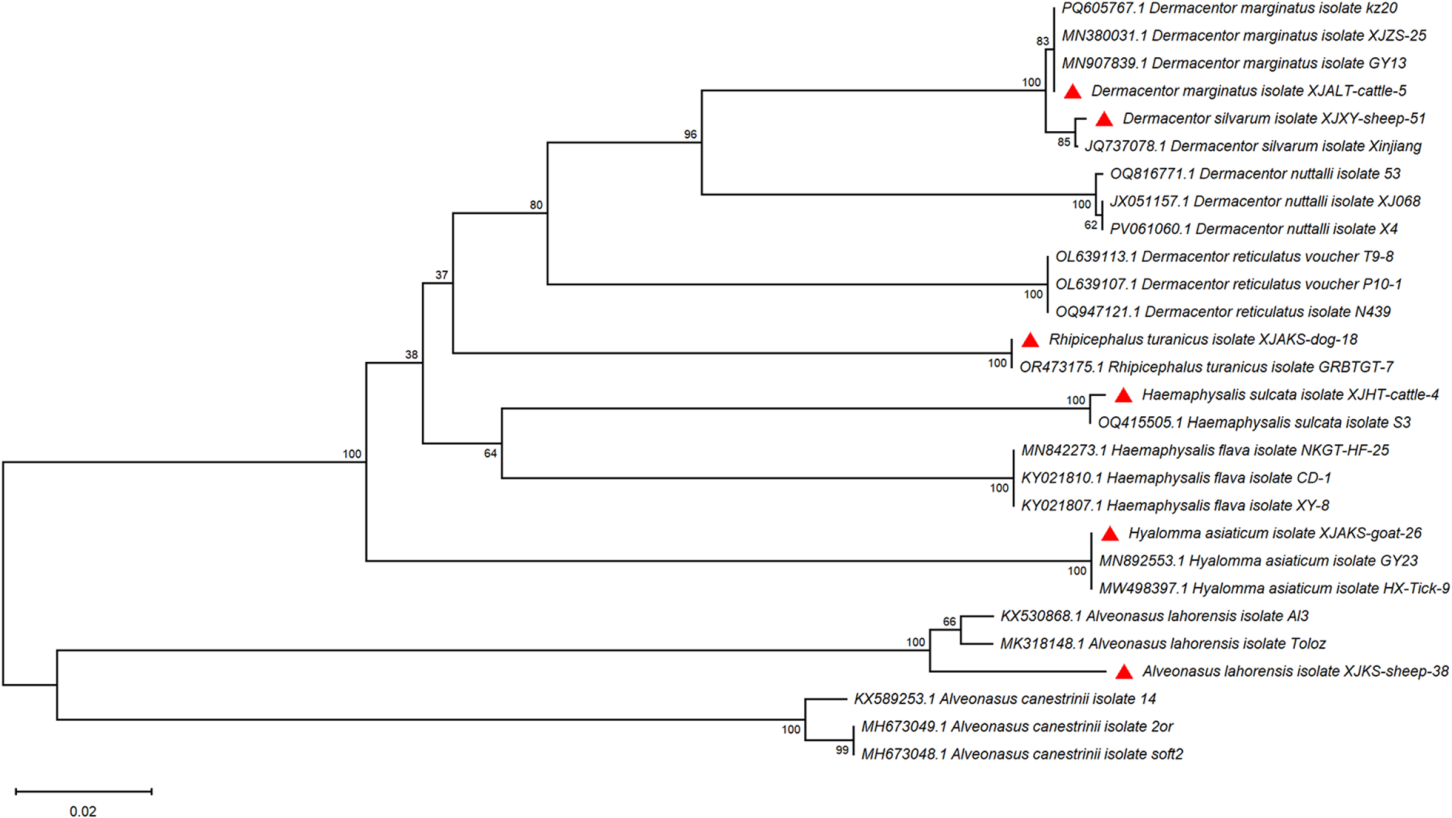
Phylogenetic analysis of ticks based on *COI* nucleotide sequences. Sequences obtained in this study are marked with red triangles before their names

Seven species across five genera were confirmed from 6172 adults: *Alveonasus lahorensis* (33.7%, 2077/6172), *Dermacentor marginatus* (32.3%, 1992/6172), *Rhipicephalus turanicus* (21.9%, 1351/6172), *Dermacentor silvarum* (5.7%, 352/6172), *Hyalomma asiaticum* (4.0%, 247/6172), and *Haemaphysalis sulcata* (2.5%, 153/6, 172). COI gene barcoding confirmed morphological identifications with > 98% sequence similarity to reference strains (Fig. 2). Species–host associations varied significantly (*χ*^2^ = 245.7, *df* = 24, *P* < 0.001). *D. marginatus* dominated large ungulates (cattle: 58.9%, sheep: 41.1%), *A. lahorensis* was predominantly found on sheep (96.2%), and *R. turanicus* exhibited the broadest host range across all livestock species (Table 3).

**Table 3:** Summary of tick species identified and their host associations

Species	Total no.	Percentage (%)	Host distribution	Counties detected
*Alveonasus lahorensis*	2077	33.7	Sheep (96.2%), cattle (3.8%)	12 counties
*Dermacentor marginatus*	1992	32.3	Sheep (58.9%), cattle (35.4%), horse (5.7%)	6 counties
*Rhipicephalus turanicus*	1351	21.9	Sheep (73.6%), goats (9.7%), dogs (9.3%), cattle (7.4%)	9 counties
*Dermacentor silvarum*	352	5.7	Sheep (100%)	1 county
*Hyalomma asiaticum*	247	4.0	Goats (100%)	1 county
*Haemaphysalis sulcata*	153	2.5	Cattle (100%)	1 county
Total	6172	100		18 counties

### Pathogen detection and prevalence

#### *Rickettsia* spp.

**Fig. 3:**
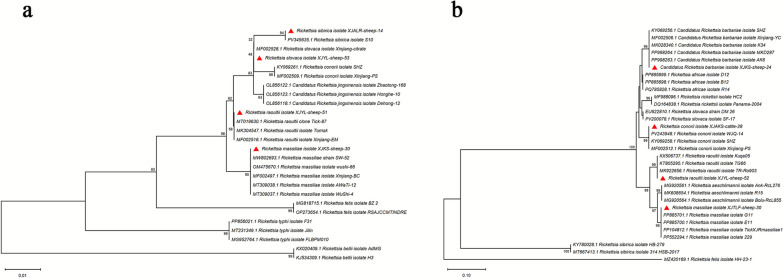
Phylogenetic analysis of *Rickettsia* strains based on the nucleotide sequences of *gltA* (**a**) and *ompA* (**b**). Sequences obtained in this study are marked with red triangles before their names

*Rickettsia* DNA was detected in 28/55 pools (50.9%; 95% confidence interval [CI]: 38.1–63.6%), representing 168 individual ticks with a minimum infection rate (MIR) of 16.7%. Phylogenetic analysis of *gltA* and *ompA* sequences revealed three distinct spotted fever group species (Fig. 3): *R. raoultii* (12 pools, 43.0%; 72 individual ticks), *R. massiliae* (10 pools, 35.7%; 60 ticks), and *R. barbariae* (6 pools, 21.4%; 36 ticks). Geographic analysis showed *R. raoultii* predominance in northern counties (Altay, Xinyuan, and Zhaosu), while *R. massiliae* was more frequent in southern regions (Kashgar, Aksu, and Awat).

#### *Anaplasma* spp.

**Fig. 4:**
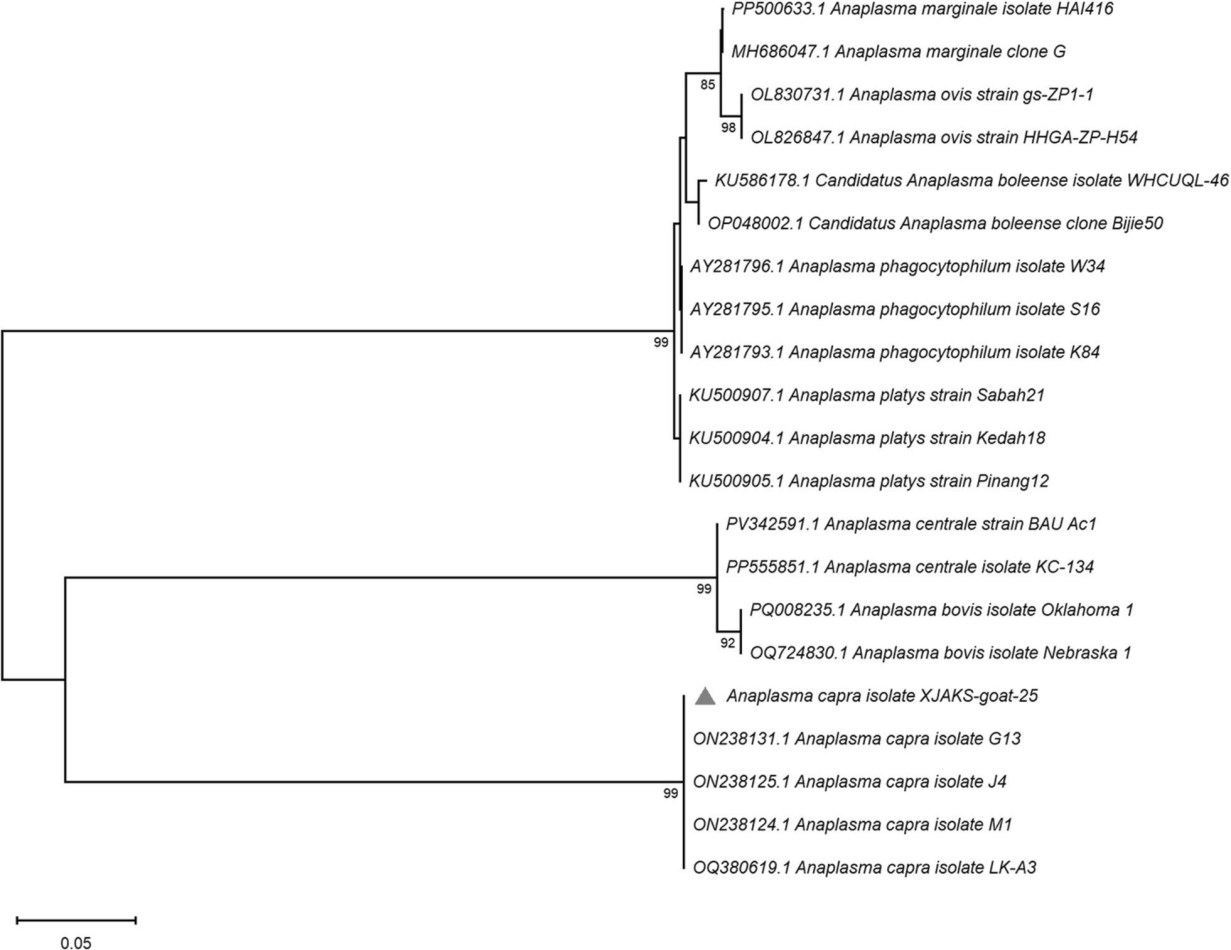
Phylogenetic tree based on nucleotide sequences of *16S rRNA* sequences of *Anaplasma* spp. Sequences obtained in this study are marked with red triangles before their names

*Anaplasma capra* was detected in one pool of *D. marginatus* collected from goats in Shaya County (1/55 pools, 1.8%; 95% CI: 0.1–9.4%), representing 6 individual ticks (MIR: 16.7%). The 16S rRNA sequence (GenBank: PV875542) showed 99.7% identity with reference *A. capra* strains and clustered with high bootstrap support (100%) in phylogenetic analysis (Fig. 4).

#### *Borrelia* spp.

**Fig. 5:**
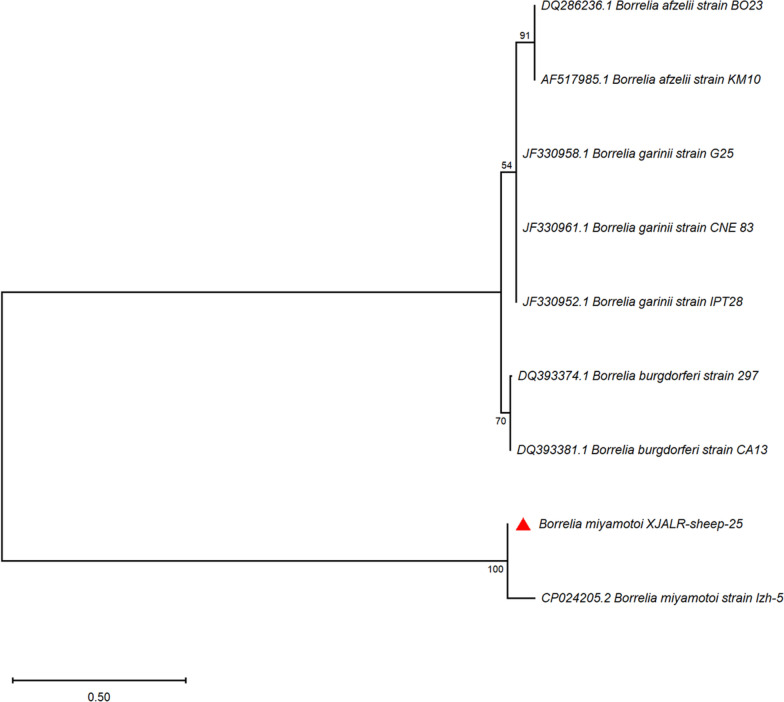
Phylogenetic tree based on nucleotide sequences of *groEL* sequences of *Borrelia* spp. Sequences obtained in this study are marked with red triangles before their names

*Borrelia miyamotoi* was identified in one pool of *R. turanicus* from sheep in Aral County (1/55 pools, 1.8%; 95% CI: 0.1–9.4%), representing six individual ticks (MIR: 16.7%). The *groEL* sequence (GenBank: PV936263) demonstrated 100% identity with *B. miyamotoi* reference strains from Asia with high phylogenetic support (99% bootstrap value; Fig. 5).

### Additional bacterial diversity

Broad-range *16S rRNA* screening identified five bacterial genera from pooled samples (Table 4): *Vibrio* species in *R. turanicus* from sheep, *Coxiella* species in *R. turanicus* from dogs, *Francisella* species in *H. asiaticum* from goats, *Arsenophonus* species in *D. marginatus* from cattle and sheep, and *Psychrobacter* species in *D. marginatus* from goats and *A. lahorensis* from sheep.

**Table 4:** Tick-borne bacterial pathogens detected in different tick species and their hosts

Pathogen species	Tick species	Host	Pooled tick group	GenBank accession no.
*Vibrio* spp.	*R. turanicus*	Sheep	6	PX129236
*Coxiella* spp.	*R. turanicus*	Dogs	17	PX129231
*Francisella* spp.	*H. asiaticum*	Goat	26	PX129237
*Arsenophonus* spp.	*D. marginatus*	Cattle	15	PX129256
	*D. marginatus*	Sheep	47	PX129257
*Psychrobacter* spp.	*D. marginatus*	Goat	27	PX129542
	*A. lahorensis*	Sheep	32	PX129541

### Evidence for vertical transmission

Laboratory-maintained *R. turanicus* females (*n* = 5) produced viable eggs that developed into larvae. *R. massiliae* DNA was detected in both egg masses (3/5, 60%; 95% CI: 14.7–94.7%) and unfed newly hatched larvae (4/6, 66.7%; 95% CI: 22.3–95.7%) using *gltA*-specific PCR. Sequences (GenBank: PX233672) showed 99.7% identity with reference *R. massiliae* strains, providing molecular evidence consistent with transovarial transmission.

## Discussion

This study provides molecular characterization of livestock-associated ticks and their bacterial pathogens in Xinjiang, northwestern China. The findings include high prevalence of spotted fever group (SFG) *Rickettsia*, detection of *Anaplasma capra* and *Borrelia miyamotoi*, and molecular evidence of vertical transmission of *Rickettsia massiliae* in *Rhipicephalus turanicus*. These results expand knowledge of pathogen circulation in pastoral ecosystems at the China–Central Asia interface and have important implications for animal and human health [16].

The pool-level prevalence of SFG *Rickettsia* detected in this study (50.9%) substantially exceeds levels reported from Inner Mongolia or Heilongjiang and is comparable to those observed in Central Asia [17, 18]. However, it should be noted that pool-based detection may overestimate individual-level infection rates compared with testing individual specimens; our calculated MIR of 16.7% represents a more conservative lower-bound estimate. The spatial distribution pattern, with *R. raoultii* predominating in northern counties and *R. massiliae* in southern areas, suggests that ecological gradients shape pathogen distribution across Xinjiang. Both species are recognized human pathogens associated with tick-borne lymphadenopathy and Mediterranean spotted fever-like illness, respectively [19, 20]. While the elevated prevalence suggests potential exposure risks in pastoral settings, the actual human health burden remains unquantified in the absence of seroepidemiological surveys or clinical surveillance data from local populations. These findings underscore the need for integrated One Health investigations that combine tick surveillance with human seroprevalence studies and clinical case detection to validate zoonotic risk assessments.

The detection of *A. capra* in *Dermacentor marginatus* from goats represents an important epidemiological observation. Since its identification in China in 2015, *A. capra* has been associated with human febrile illness across Asia, establishing its zoonotic potential [21–23]. The widespread distribution of goats in Xinjiang and their frequent interaction with humans provide a plausible pathway for zoonotic transmission [24, 25], though human infection data from this region are currently lacking. The low detection rate (1.8% pool positivity, one positive pool from 55 tested) limits robust inference regarding true prevalence. Nevertheless, detection warrants attention given that *A. capra* infections may be underdiagnosed owing to nonspecific clinical symptoms [26].

The detection of *B. miyamotoi* in *R. turanicus* from sheep extends the known distribution of this relapsing fever spirochete within China [27], though the single positive pool (1.8% of samples) precludes robust assessment of true prevalence in this region. Unlike *Borrelia burgdorferi* sensu lato, which causes Lyme borreliosis, *B. miyamotoi* is increasingly recognized as a human pathogen responsible for hard tick-borne relapsing fever [28, 29]. Its presence in Xinjiang tick populations suggests local transmission may occur, though the actual human disease incidence remains unknown owing to absence of systematic clinical surveillance. Given its clinical overlap with other infections, misdiagnosis may occur, reinforcing the necessity for comprehensive epidemiological studies in both human and livestock populations [30].

Beyond these recognized pathogens, several bacterial genera were identified, including *Arsenophonus*, *Psychrobacter*, *Coxiella*, *Vibrio*, and *Francisella*. While some may function as endosymbionts, others could influence tick physiology or pathogen interactions [31]. Their ecological significance remains to be elucidated, but their detection reflects the complexity of tick microbiomes [32]. Future metagenomic approaches will be essential to clarify their roles and interactions with pathogenic species [33].

A notable finding is the detection of *R. massiliae* DNA in eggs and unfed larvae of *R. turanicus*, consistent with transovarial transmission. The detection rates in eggs (60%) and larvae (66.7%) suggest vertical transmission may occur, though important limitations must be considered. First, the small sample size (*n* = 5 females) produces wide confidence intervals and limits precise estimation. Second, DNA detection does not confirm viable, infectious bacteria; culture-based studies would be required. Third, we assessed only first-generation transmission; efficiency in subsequent generations remains unknown. Despite these caveats, our findings suggest transovarial transmission of *R. massiliae* in *R. turanicus* may occur, though confirmation requires culture-based viability studies and assessment of transmission efficiency across multiple generations. If confirmed, vertical transmission could partially contribute to the observed prevalence, though horizontal acquisition through blood meals likely remains predominant [34].

Overall, these findings highlight the importance of integrated One Health surveillance in Xinjiang, where livestock act as tick hosts and potential amplifiers of zoonotic pathogens. Enhanced animal-based surveillance could serve as an early warning system, though this requires validation through parallel human surveillance. Cross-border collaboration is particularly critical given Xinjiang’s position at the China–Central Asia interface, where livestock trade may facilitate pathogen movement [9].

Several important limitations must be acknowledged when interpreting these findings. First, the pooling strategy introduces bidirectional bias: while cost-effective for initial screening, it may underestimate true infection rates for pathogens present at low individual-level prevalence owing to dilution effects within pools [35]. Second, the cross-sectional design also restricted assessment of seasonal variation, which may influence circulation patterns. Third, pathogen DNA detection alone cannot confirm vector competence, and experimental transmission studies are required to establish epidemiological relevance.

## Conclusions

Seven tick species parasitize livestock in Xinjiang, with three dominant taxa accounting for > 85% of specimens. Sequence-confirmed detection of spotted fever group *Rickettsia*, *A. capra*, and *B. miyamotoi* highlights the circulation of zoonotic pathogens in pastoral settings. Importantly, molecular evidence for vertical transmission of *R. massiliae* demonstrates efficient maintenance in tick populations. Together, these findings provide a comprehensive molecular baseline for northwestern China and emphasize the critical need for strengthened One Health surveillance to mitigate zoonotic disease risks in pastoral communities.

## Data Availability

Representative sequences have been deposited in GenBank under accession nos. PV875509–PV875515 (*COI* tick sequences), PV936264–PV932627 (*gltA Rickettsia* sequences), PV936268–PV932671 (*ompA Rickettsia* sequences), PV875542 (16S rRNA *Anaplasma* sequences), and PV936263 (*groEL Borrelia* sequences).

## Abbreviations

*COI*: Cytochrome oxidase I
*gltA*: Glutamate transporter A
*ompA*: Outer membrane protein A
rRNA: Ribosomal RNA
*groEL*: Heat shock protein GroEL
PCR: Polymerase chain reaction
SFG: Spotted fever group
MIR: Minimum infection rate
CI: Confidence interval
PBS: Phosphate-buffered saline
